# Hemodynamic Versus Anatomic Assessment of Symptomatic Atherosclerotic Middle Cerebral Artery Stenosis: the Relationship Between Pressure Wire Translesional Gradient and Angiographic Lesion Geometry

**DOI:** 10.3389/fneur.2021.671778

**Published:** 2021-08-11

**Authors:** Long Li, Bin Yang, Adam A. Dmytriw, Tao Wang, Jichang Luo, Yanling Li, Yan Ma, Jian Chen, Yabing Wang, Peng Gao, Yao Feng, Xuesong Bai, Xiao Zhang, Jia Dong, Renjie Yang, Liqun Jiao, Feng Ling

**Affiliations:** ^1^Department of Neurosurgery, Xuanwu Hospital, Capital Medical University, Beijing, China; ^2^Neuroradiology & Neurointervention Service, Brigham and Women's Hospital, Harvard Medical School, Boston, MA, United States; ^3^Department of Epidemiology and Biostatistics, School of Public Health, Capital Medical University, Beijing, China; ^4^Department of Interventional Neuroradiology, Xuanwu Hospital, Capital Medical University, Beijing, China

**Keywords:** intracranial cerebral atherosclerosis, stenosis, hemodynamics, translesional pressure gradient, Pd/Pa, Pa-Pd

## Abstract

**Background:** Intracranial cerebral atherosclerosis (ICAS) is a leading etiology of ischemic stroke. The diagnosis and assessment of intracranial stenosis are shifting from anatomic to hemodynamic for better risk stratification. However, the relationships between lesion geometry and translesional pressure gradient have not been clearly elucidated.

**Methods:** Patients with symptomatic unifocal M1 middle cerebral artery (M1-MCA) stenosis were consecutively recruited. The translesional pressure gradient was measured with a pressure wire and was recorded as both mean distal/proximal pressure ratios (Pd/Pa) and translesional pressure difference (Pa–Pd). Lesion geometry measured on angiography was recorded as diameter stenosis, minimal lumen diameter, and lesion length. The correlations between pressure-derived and angiography-derived indices were then analyzed.

**Results:** Forty-three patients were analyzed. A negative correlation was found between Pd/Pa and diameter stenosis (*r* = −0.371; *p* = 0.014) and between Pa – Pd and minimal lumen diameter (*r* = −0.507; *p* = 0.001). A positive correlation was found between Pd/Pa and minimal lumen diameter (*r* = 0.411; *p* = 0.006) and between Pa – Pd and diameter stenosis (*r* = 0.466; *p* = 0.002).

**Conclusions:** In a highly selected ICAS subgroup, geometric indices derived from angiography correlate significantly with translesional pressure gradient indices. However, the correlation strength is weak-to-moderate, which implies that anatomic assessment could only partly reflect hemodynamic status. Translesional pressure gradient measured by pressure wire may serve as a more predictive marker of ICAS severity. More factors need to be identified in further studies.

## Introduction

Intracranial cerebral atherosclerosis (ICAS) is the most common cause of ischemic events worldwide, particularly in Asian, Hispanics, and Africans, and may be underestimated in Caucasians (1–4). In the Warfarin-Aspirin Symptomatic Intracranial Disease (WASID) trial, higher degrees of anatomic stenosis were identified as independent predictors of recurrent ischemic stroke (5, 6). This inspired investigators to adopt more aggressive treatments, including balloon angioplasty or stenting, toward improved outcomes in patients with >70% stenosis. However, failures of previous randomized controlled trials have raised the concern that there may be bias in identifying patients at high risk solely on anatomic assessment (7–9). Additional approaches are thus urgently needed.

In ICAS patients with ischemic stroke, identification of the underlying mechanism is critical for management. The current proposed mechanisms include artery-to-artery embolism, perforator occlusion, hemodynamic dysfunction, and mixed etiologies (10). Patients with symptomatic ICAS and hemodynamic insufficiency may benefit from angioplasty beyond optimal medical therapy alone, as the former could further improve distal perfusion (11–13). Thus, hemodynamic assessment may improve stratification of patients for such treatment strategies. Hemodynamic insufficiency may be inferred from infarct pattern (i.e., watershed infarction) or various models of cerebral perfusion imaging (e.g., asymmetry between bilateral hemispheres). However, these are ultimately evaluations of brain parenchyma, and focal or arterial lesion-related assessments of hemodynamics could have more important therapeutic implications.

Fractional flow reserve (FFR), defined as the ratio of maximum flow in the presence of a stenosis to normal maximum flow, has become the gold standard in assessing the hemodynamic insufficiency of epicardial coronary stenosis (14–16). Besides, translesional pressure gradient and its derivative indices, for instance, rest Pd/Pa and instantaneous wave-free ratio (iFR), have been widely used on guiding coronary revascularization therapy (17–20). Basically, they utilize a pressure wire to measure mean distal coronary (Pd) and arterial pressure (Pa) with or without pharmacological vasodilation and present as their radio (Pd/Pa). It has demonstrated that FFR and these lesion-related indices are superior on defining myocardial ischemia risk than the degree of coronary stenosis.

Despite that hemodynamic assessment by pressure wire has been well-established in coronary revascularization, the application of this invasive method in ICAS has rarely been explored. A few studies validated its feasibility (21–24); however, the difference and relationships between pressure-derived and angiography-derived indices have not been fully elucidated. In the current study, we preliminarily investigated the correlations in a selected group of ICAS patients.

## Materials and Methods

### Patient Selection

From June 2019 to December 2020, patients that were scheduled for digital subtraction angiography (DSA) for symptomatic intracranial atherosclerosis stenosis were consecutively recruited to an observational study in our quaternary center. The study protocol was approved by the local Institutional Review Board, and all patients provided written informed consent.

Adult patients meeting the following criteria were enrolled for the current study: (1) presented with recurrent stroke or transient ischemic attack (TIA) within the past 6 months attributed to 50–99% unifocal M1 middle cerebral artery (M1-MCA) stenosis; (2) patients understood that the pressure measurements were part of a novel functional assessment of MCA stenosis for study purposes and give permission to the off-label use of a pressure wire; and (3) expected ability to traverse the lesion with a pressure wire. We excluded patients based on the following criteria: (1) non-atherosclerotic MCA stenosis (e.g., dissection, moyamoya disease, or vasculitis); (2) concurrent >50% stenosis or occlusion of intra- or extracranial arteries; (3) history of surgical and/or interventional procedures of intra- and extracranial arteries; (4) massive cerebral infarction (>1/2 MCA territory); or (5) known intracranial tumor, infection, hydrocephalus, aneurysm, or arteriovenous malformation.

### Cerebrovascular Angiography and Intracranial Pressure Measurements

General anesthesia was employed in all cases. A 6F sheath was used to access the femoral artery, after which a 6F guiding catheter was positioned in the ipsilateral petrous or foraminal internal carotid artery (ICA) segment with a 0.035-inch guidewire. Three-dimensional DSA was obtained for selecting the optimal view to access lesion geometry, including minimal lumen diameter, lesion length, and diameter stenosis. The diameter stenosis grade was calculated by measuring the minimal lumen diameter at the most stenotic site compared with the normal proximal segment diameter (first choice) or the normal feeding artery diameter (second choice), as the modified WASID method recommended in 2009 (25).

**Figure 1:**
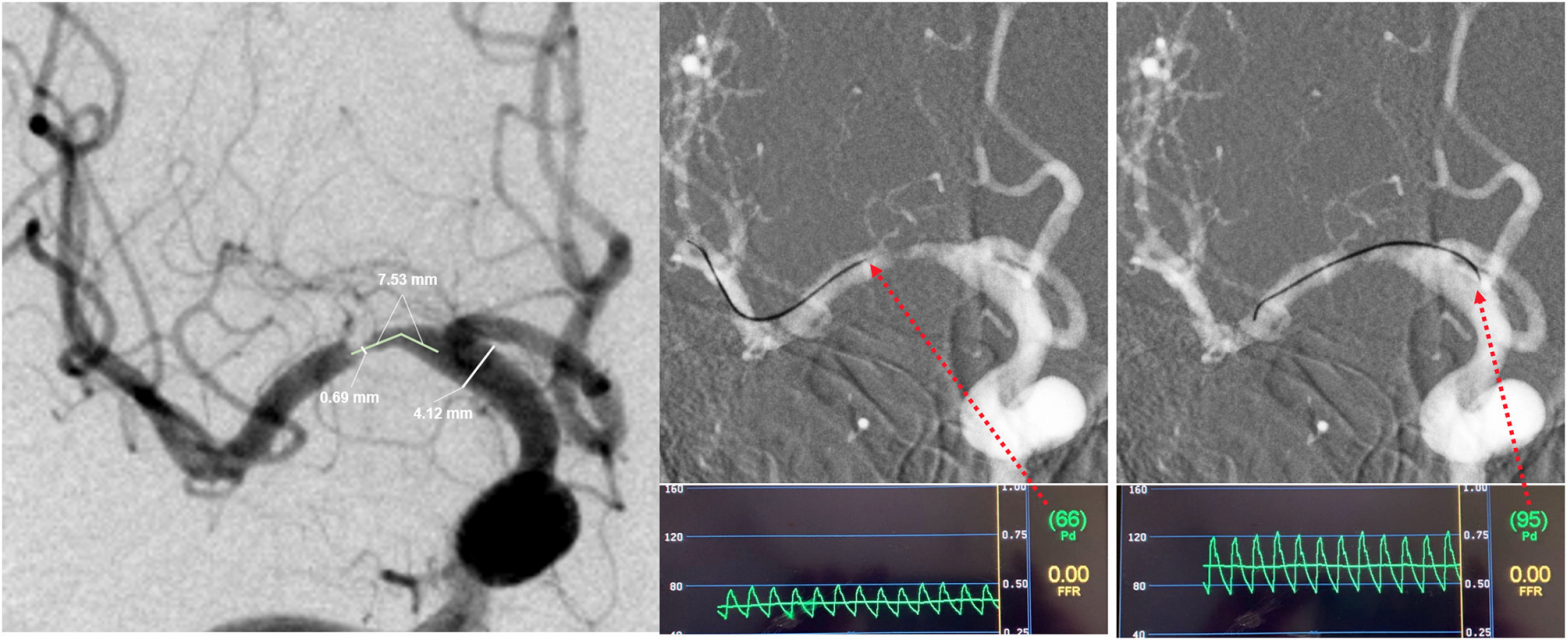
Representative case of intracranial lesion geometry and translesional pressure measurements. In this patient, **(A)** the minimal lumen diameter was 0.69 mm, and the normal segment diameter was 4.12 mm, so the stenosis was calculated as 83.3%, and the lesion length was 7.53 mm; **(B)** the mean distal pressure (Pd) was 66 mmHg; **(C)** the mean proximal pressure (Pa) was 95 mmHg. The translesional pressure gradient radio (Pd/Pa) and translesional pressure gradient difference (Pa–Pd) were thus found to be 0.69 and 29 mmHg.

A 0.014-inch pressure wire (C12008, Abbot St. Jude Medical, Minneapolis, MN, USA), designed for coronary systems, was used off-label for intracranial pressure measurements. For safety, a microcatheter (Rebar18, eV3 Covidien, Irvine, CA, USA) was used by exchange technique in all procedures to aid the pressure wire crossing the stenosis under roadmap guidance. Mean distal pressure (defined as Pd in current study) and proximal pressure (defined as Pa) were measured successively under resting conditions. The translesional pressure gradient radio (Pd/Pa) and translesional pressure gradient difference (Pa–Pd) were calculated. A representative case is displayed in Figure 1.

In addition to direct correlation analysis, subgroup analysis based on stenosis severity classification and collateral circulation was also performed. Patients were divided into moderate (50–69%) and severe (70–99%) stenosis, as the SAMMPRIS did (12). And collateral circulation was evaluated using the American Society of Interventional and Therapeutic Neuroradiology/Society of Interventional Radiology (ASITN/SIR) collateral flow grading system (26). Patients with grades 0–2 were grouped as poor collateral circulation, and grades 3–4 were deemed good ones.

### Intra-Observer and Inter-Observer Variability

Two experienced neurointerventionalists (first observer [LL] and second observer [BY]), who were blinded to clinical data and pressure values, measured angiographic geometry of all lesions independently. To assess intra-observer variability, the first grader repeated the step 4 weeks after an initial measurement again.

### Statistical Analyses

Intra- and inter-observer measurements of minimal lumen diameter, diameter stenosis, and lesion length were evaluated by absolute agreement model of intraclass correlation coefficient (ICC) analyses. Quantitative data were examined to determine presence of normal distribution. Continuous variables of normally distributed were expressed as mean ± standard deviation, or median [interquartile range (IQR)] in the presence of abnormal distribution, and qualitative data were described as percentages. The correlations between Pd/Pa or Pa–Pd and stenosis and lesion length were assessed by Pearson's test for normally distributed continuous variables while Spearman's test for non-normally distributed variables. All statistical analyses were carried out in SPSS version 23.0 and R studio. All reported *p*-values were two-sided, and *p* < 0.05 was considered to indicate statistical significance.

## Results

### Demographics

Proximal and distal pressure datasets were obtained in 43 consecutive patients after angiography. There were no complications related to translesional pressure measurement. Mean age was 54 ± 10 years, and 31 (72.1%) were male. The qualifying event was recurrent stroke in 30 patients (69.8%) and recurrent TIA in the other 13 patients (30.2%). The mean angiographic percentage of stenosis and minimal lumen diameter were 75.9 ± 7.8% and 0.59 ± 0.22 mm, respectively. The mean lesion length was 6.81 (median, 5.90; IQR, 4.78–8.62) mm. Patient characteristics and vascular risk factors are summarized in Table 1.

**Table 1:** Clinical characteristics and lesion profile.

**Variables**	***N* = 43**
Age, mean (±SD), years	54 (±10)
Male, *n* (%)	31 (72.1)
BMI, mean (±SD)	25.7 (±2.7)
Hypertension, *n* (%)	31 (72.1)
Diabetes mellitus, *n* (%)	14 (32.6)
Hyperlipidemia, *n* (%)	16 (37.2)
Coronary heart disease, *n* (%)	3 (7.0)
Atrial fibrillation, *n* (%)	0
Tobacco use, *n* (%)	22 (51.2)
Alcohol use, *n* (%)	20 (46.5)
Qualifying event—stroke, *n* (%)	30 (69.8)
Qualifying event—TIA, *n* (%)	13 (30.2)
Diameter stenosis, mean (±SD), %	75.9 (±7.8)
Diameter stenosis-−50–69%, *n* (%)	6 (14.0)
Diameter stenosis-−70–99%, *n* (%)	37 (86.0)
Minimal lumen diameter, mean (±SD), mm	0.59 (±0.22)
Lesion length, median (IQR), mm	5.90 (4.78–8.62)
Resting Pd/Pa, median (IQR)	0.69 (0.49–0.74)
Pa – Pd, mean (±SD), mmHg	28 (±13)
Collateral grading (ASITN/SIR), *n* (%)	
Grade 0	1(2.3)
Grade 1	15 (34.9)
Grade 2	1 (2.3)
Grade 3	2 (4.7)
Grade 4	24 (55.8)

*BMI, body mass index; TIA, transient ischemic attack; IQR, interquartile range; ASITN/SIR, the American Society of Interventional and Therapeutic Neuroradiology/Society of Interventional Radiology collateral flow grading system*.

### Correlation Between Pressure-Derived and Angiography-Derived Indices

**Figure 2:**
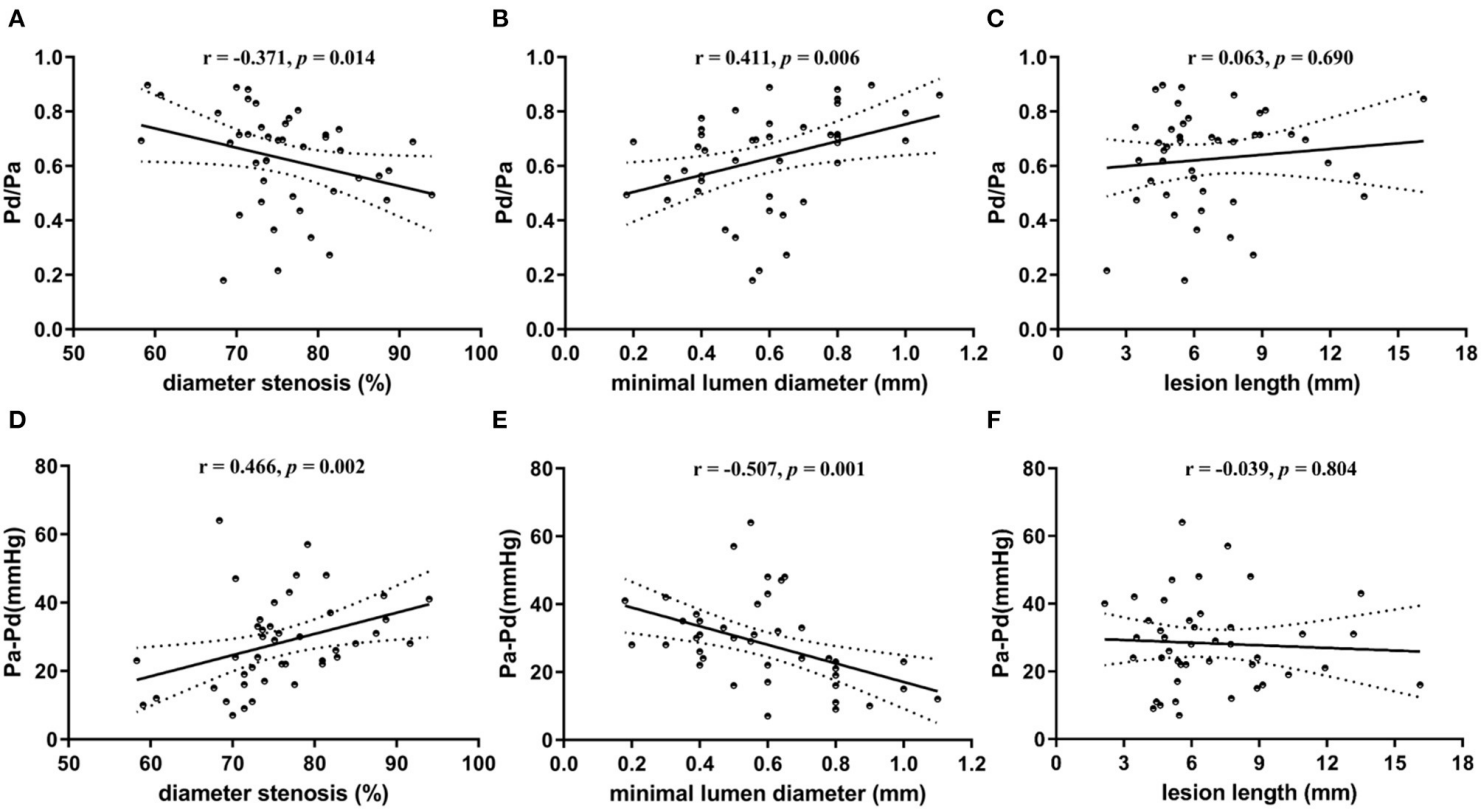
Scatter plots showing correlation between translesional pressure gradient and lesion geometry. **(A)** Pd/Pa vs. diameter stenosis; **(B)** Pd/Pa vs. minimal lumen diameter; **(C)** Pd/Pa vs. lesion length; **(D)** Pa-Pd vs. diameter stenosis; **(E)** Pa-Pd vs. minimal lumen diameter; **(F)** Pa-Pd vs. lesion length. Pd indicates distal pressure of stenosis; and Pa indicates proximal pressure of stenosis. ※ indicates Spearman test for non-normally distributed variables; and ^†^ indicates Pearson test for normally distributed continuous variables.

Regarding *correlation between resting Pd/Pa and lesion geometry*, the median resting Pd/Pa was 0.69 (IQR 0.49–0.74). A weak but statistically significant correlation was found between resting Pd/Pa and diameter stenosis (*r* = −0.371; *p* = 0.014; Figure 2A). The correlation was stronger between resting Pd/Pa and minimal lumen diameter (*r* = 0.411; *p* = 0.006; Figure 2B). No significant correlation was found between resting Pd/Pa and lesion length (Figure 2C).

Regarding *correlation between translesional pressure gradient and lesion geometry*, the translesional pressure gradient (Pa–Pd) ranges from 7 to 64 (mean 28 ± 13) mmHg. Compared with resting Pd/Pa, it presented further enhanced correlations with both diameter stenosis (*r* = 0.466; *p* = 0.002; Figure 2D) and minimal lumen diameter (*r* = −0.507; *p* = 0.001; Figure 2E). This was also uncorrelated with lesion length (Figure 2F).

Statistically, the strongest correlation was between Pa–Pd and minimal lumen diameter (correlation coefficients: 0.507).

### Correlation Between Pressure-Derived and Angiography-Derived Indices Based on Stenosis Severity Classification and Collateral Circulation

#### Subgroup Analysis Based on Stenosis Severity Classification

Six patients (14%) were found to have moderate stenosis. There was no significant difference in patients with moderate and severe stenoses on neither Pd/Pa (0.69 ± 0.26 vs. 0.62 ± 0.17, *p* = 0.107) nor Pa – Pd (22.5 ± 20.1 vs. 29.2 ± 11.5 mmHg, *p* = 0.248).

In the moderate stenosis group, no significant correlation was found between pressure-derived and angiography-derived indices. In the severe stenosis group, statistically significant correlations were found between resting Pd/Pa and diameter stenosis (*r* = −0.341; *p* = 0.039), and between Pa–Pd and diameter stenosis (*r* = 0.352; *p* = 0.033) and minimal lumen diameter (*r* = −0.343; *p* = 0.038). The detailed results are presented in Table 2.

**Table 2:** Correlation between pressure-derived and angiography-derived indices based on stenosis severity classification.

	**Moderate stenosis (50–69%)**				**Severe stenosis (70–99%)**			
	**Resting Pd/Pa**		**Pa–Pd (mmHg)**		**Resting Pd/Pa**		**Pa–Pd (mmHg)**	
	***r***	***p***	***r***	***p***	***r***	***p***	***r***	***p***
**Minimal lumen diameter (mm)**	0.638	0.173	−0.116	0.827	0.286	0.087	−0.343	0.038
**Diameter stenosis (%)**	−0.600	0.208	0.029	0.957	−0.341	0.039	0.352	0.033
**Lesion length (mm)**	0.143	0.787	0.486	0.329	0.051	0.764	−0.078	0.645

#### Subgroup Analysis Based on Collateral Circulation

Seventeen patients (39.5%) were grouped as poor collateral circulation status, and the other 26 (60.5%) were grouped as good. The correlation between pressure-derived and angiography-derived indices was still significant in patients with good collateral circulation. However, no significant correlation was found in patients with poor collateral circulation. The detailed results are presented in Table 3.

**Table 3:** Correlation between pressure-derived and angiography-derived indices based on collateral circulation.

	**Poor collateral (ASITN/SIR 0–2)**				**Good collateral (ASITN/SIR 3–4)**			
	**Resting Pd/Pa**		**Pa–Pd (mmHg)**		**Resting Pd/Pa**		**Pa–Pd (mmHg)**	
	***r***	***p***	***r***	***p***	***r***	***p***	***r***	***p***
**Minimal lumen diameter (mm)**	0.318	0.213	−0.591	0.012	0.462	0.018	−0.492	0.011
**Diameter stenosis (%)**	−0.129	0.622	0.327	0.200	−0.469	0.016	0.538	0.005
**Lesion length (mm)**	0.105	0.687	−0.115	0.659	−0.069	0.736	0.083	0.687

*ASITN/SIR, the American Society of Interventional and Therapeutic Neuroradiology/Society of Interventional Radiology collateral flow grading system*.

### Intra-Observer and Inter-Observer Variability

Intra-observer measurements of lesion geometry revealed ICC values of 0.966, 0.976, and 0.955 for minimal lumen diameter, diameter stenosis, and lesion length, respectively, which indicate near-perfect agreement (Table 4). Meanwhile, inter-observer measurements revealed ICC values of 0.870, 0.842, and 0.902 for minimal lumen diameter, diameter stenosis, and lesion length respectively, which indicate good agreement (Table 4).

**Table 4:** Intra-and inter-observer variability analyses of lesion geometry.

	**Minimal lumen diameter** **(mm)**		**Diameter stenosis** **(%)**		**Lesion length** **(mm)**	
	**ICC (95% CI)**	***p***	**ICC (95% CI)**	***p***	**ICC (95% CI)**	***p***
Intra-observer	0.966 (0.937, 0.981)	<0.001	0.976 (0.956, 0.987)	<0.001	0.955 (0.951, 0.957)	<0.001
Inter-observer	0.870 (0.774, 0.927)	<0.001	0.842 (0.726, 0.911)	<0.001	0.902 (0.831, 0.921)	<0.001

*Intra-observer and inter-observer variability and agreement were evaluated using absolute agreement model of ICC analyses. CI, confidence interval; ICC, intraclass correlation coefficient*.

## Discussion

The present study demonstrated that in patients with symptomatic M1 stenosis, hemodynamic indices as assessed by pressure wire are significantly associated with anatomic indices on DSA. This was reflected by weak-to-moderate correlations between translesional pressure gradient measured by pressure wire (Pd/Pa and Pa–Pd) and lesion geometry measured by angiography (diameter stenosis and minimal lumen diameter). Our interpretation is that either luminal diameter or percentage stenosis only partly reflects hemodynamic status, and stenoses require greater assessment to evaluate the hemodynamic functional significance of intracranial atherosclerotic stenosis. Translesional pressure gradient may serve as a more predictive marker of ICAS severity.

A few studies have investigated the relationship between luminal narrowing and hemodynamic indices, measured by pressure wire in ICAS, without consensus reached as yet on its utility. Mario et al. reported that that neither luminal diameter nor percentage stenosis (visual or quantified) was correlated with distal/proximal pressure ratios or proximal-to-distal pressure gradients (23), which is contrary to our findings. The difference may be due to the small sample size and various lesion sites (*n* = 9: 2 cavernous, three supraclinoid, and 4 M1) in their study.

Liu et al. demonstrated a weak-to-moderate correlation between anatomic stenosis and hemodynamic measurements across an atherosclerotic lesion (22), which is consistent with our study. However, the authors emphasized that this only reached statistical significance in patients with poor collateralization, which implies that collateral state may be another factor that affects translesional pressure gradient in ICAS, which was not shown in our study. There may be several reasons for the difference. First, the sample size of both Liu's (*n* = 25) and our study (*n* = 43) was limited. Therefore, the results of subgroup analysis were unreliable. Second, the current grading system, for instance, the ASITN/SIR grade (26), the Capillary Index Score (CIS) (27), or the MMD Collateral Grading System (28), may have inadequate accuracy to grade the hemodynamic status for this application. A novel composite grading system, such as the angiographic DILEMMA score for cardiovascular disease (29), which combines lesion geometry indices and dynamic changes in angiography, could be more powerful. In addition, this score requires the inclusion of less-selected patients.

It should be noted that an accurate and precise measurement of the lesion geometry is critical for meaningful assessment of ICAS or other hemodynamic analyses. For stenosis grading of the intracranial major cerebral arteries, the WASID Group established a reliable method in 1999 (30), which is now widely used in clinical practice (22). For the denominator, the normal proximal segment, distal segment, and feeding artery diameter are chosen as the first, second, and third options, respectively. However, to avoid the error caused by slight physiologic distal narrowing of some intracranial arteries, we always choose proximal diameter as the denominator (i.e., the normal proximal segment diameter and feeding artery diameter as the first and second choices, respectively) (25). Furthermore, for circumventing the limitation on choosing a denominator, we also compared the minimal luminal diameter as an independent index with accompanying pressure-derived indices. Our findings suggest that this method may be more accurate than grade of stenosis alone.

The relationship between pressure gradient and lesion geometry was also studied in interventional cardiology literature. The results appear to vary according to the population included. A curvilinear relation was found by Jozef et al. between myocardial FFR and both stenosis diameter (*r* = 0.79) and the smallest lumen diameter (*r* = 0.82) (31). Osman et al. found a significant linear correlation between FFR and lesion length (*r* = −0.314) and minimal lumen diameter (*r* = 0.415) (29). Antonio et al. demonstrated that FFR values were correlated to the smallest lumen diameter (*r* = 0.34) and stenosis diameter (*r* = −0.28) (32). Compared with the coronary arteries, intracranial arteries have more intricate collateral circulation (i.e., the circle of Willis and secondary collateral pathways) and cerebral blood flow autoregulation. In addition, blood pressure, lesion morphology, and characteristics, as well as the territory perfused, may also affect the hemodynamic state of cerebral arteries. In ICAS, the application of hemodynamic assessment is still in its naissance and requires further exploratory study and evidence.

Impediments to replicating the success of focal hemodynamic assessment in cardiovascular disease in the context of ICAS partly lie in the lack of well-recognized and reliable evaluation methods. For instance, there is no similar gold standard to exercise testing to indicate inducible ischemia in cerebrovascular field (33). Xenon computed tomography (Xe-CT), the gold standard method to quantitatively measure cerebral blood flow, is difficult to obtain and intolerant for a considerable proportion of patients (34). Nevertheless, an increasing number of stroke clinicians are beginning to shift their focus from intracranial stenosis grading to hemodynamic assessment.

Some researchers have attempted to establish the relations between hemodynamic indices and stroke risk by non-invasive approaches, with promising results. The modalities include signal intensity ratio (SIR) on time-of-flight magnetic resonance angiography, computational fluid dynamics (CFD) modeling, and quantitative magnetic resonance angiography (QMRA) (35–41). But all have their own limitations. We assume that direct translesional pressure gradient measurement could play a leading role. And large and prospective cohort studies have been started, which may provide stronger evidence.

### Study Limitations

Limitations of the current study include the following: (1) the findings are based on a highly selected group. In patients with symptomatic unifocal M1 stenosis, the factors affecting hemodynamic status may be limited to blood pressure, secondary collateral circulation (i.e., anastomoses from ipsilateral anterior cerebral artery and posterior cerebral artery to MCA supply) and distal territory resistance. Thus, the correlation may alter as the lesion site changes. For instance, when lesions are in the terminal segment of ICA (distal to posterior communicating artery) or middle segment of ICA (proximal to posterior communicating artery), blood flow may vary through the circle of Willis. (2) Unlike the measurement of FFR in coronary stenosis, we measured the pressure without the use of vasodilatory agents, as previous studies have demonstrated that intracranial arteries are often already maximally dilated in symptomatic patients (42, 43). Based on this, we further simplified the process of pressure measurement as we equalized the pressure sensor to zero only once before introducing the pressure wire into the target vessel. Thereafter, we measured Pd and Pa successively by pullback of the pressure wire. We assumed the pressure dataset was recorded under the same condition and that its accuracy was not affected even if there was a so-called “drift” of the pressure values. Further tests are needed to verify this approach.

## Conclusions

This observational study preliminary indicates that in patients with unifocal M1 stenosis, geometry indices derived from angiography correlate significantly with translesional pressure gradient indices. However, the correlation strength is weak-to-moderate, which implies that anatomic assessment could only partly reflect the hemodynamic status. Translesional pressure gradients measured by pressure wire may serve as a more predictive marker of ICAS severity. More factors need to be identified in further studies.

## Data Availability Statement

The raw/processed data required to reproduce these findings cannot be shared at this time as the data also forms part of an ongoing study. Requests to access the datasets should be directed to the corresponding author.

## Ethics Statement

The study was conducted according to the guidelines of the Declaration of Helsinki and approved by the Institutional Review Board of Xuanwu Hospital (protocol code (20170613) and date of approval (20171011)). The patients/participants provided their written informed consent to participate in this study.

## Author Contributions

FL and LJ: conceptualization, YM, BY, YL, and LL: methodology, YL: formal analysis, JL, YM, JC, YW, PG, YF, XB, XZ, JD, and RY: data curation, LL: writing—original draft preparation, TW and AD: writing—review and editing, FL: supervision, LJ: project administration and funding acquisition. All authors have read and agreed to the published version of the manuscript.

## Conflict of Interest

The authors declare that the research was conducted in the absence of any commercial or financial relationships that could be construed as a potential conflict of interest. The handling editor HQZ declared a shared affiliation, though no other collaboration, with one or more authors LL, BY, TW, JL, YL, YM, JC, YW, PG, YF, XB, XZ, JD, RY, LJ, FL at the time of the review.

## Publisher's Note

All claims expressed in this article are solely those of the authors and do not necessarily represent those of their affiliated organizations, or those of the publisher, the editors and the reviewers. Any product that may be evaluated in this article, or claim that may be made by its manufacturer, is not guaranteed or endorsed by the publisher.
